# Evaluation of Truncated NhhA Protein as a Candidate Meningococcal Vaccine Antigen

**DOI:** 10.1371/journal.pone.0072003

**Published:** 2013-09-06

**Authors:** Ian R. Peak, Yogitha N. Srikhanta, Vincent E. Weynants, Christiane Feron, Jan T. Poolman, Michael P. Jennings

**Affiliations:** 1 Institute for Glycomics, Griffith University, Gold Coast Campus, Gold Coast, Queensland, Australia; 2 School of Molecular and Microbial Science, The University of Queensland, Brisbane, Queensland, Australia; 3 GlaxoSmithKline Vaccines, Rixensart, Belgium; Instituto Butantan, Brazil

## Abstract

NhhA (*Neisseria hia* homologue) is an outer membrane protein from *Neisseria meningitidis*, the causative agent of meningococcal disease. The protein is surface exposed and its expression in a wide range of meningococcal strains suggests it is a promising vaccine candidate. In addition, immunization of mice with outer membrane vesicles of strains that overexpress NhhA in conjunction with one of TbpA, Omp85 or NspA results in synergistic bactericidal responses. We previously showed that the NhhA sequence is highly conserved between strains, with the majority of the differences localized to four distinct variable regions located in the amino-terminal region of the mature protein. In this study, *N. meningitidis* strains were constructed that over-express wild-type NhhA. Strains expressing truncated versions of NhhA, with deletions from the amino-terminal region that removed the most variable regions, were also made. These expression strains were also modified so that immunodominant, phase- and antigenically-variable outer membrane proteins were not expressed, truncated lipooligosaccharide (LOS) expression was genetically fixed (no phase variability), and capsular polysaccharide expression abolished. Outer membrane vesicles derived from these strains were used to immunize mice. As previously observed, a synergistic effect involving another antigen, TbpA, was required to demonstrate bactericidal activity. The highest bactericidal response against a heterologous strain was obtained with a truncated variant of NhhA. These results indicate that removal of (a) variable region(s) does not reduce bactericidal responses against NhhA, and that bactericidal targets exist in regions other than the variable N-teminus. This provides the basis for future examination of responses against truncated NhhA in protecting against heterologous NhhA strains, and further evaluation of truncated NhhA as a candidate for inclusion in a vaccine against all serogroups of N. meningitidis.

## Introduction

*Neisseria meningitidis* is the causative agent of meningococcal meningitis and septicaemia. Its only known host is the human, and it may be carried asymptomatically by approximately 10% of the population [1]. There is currently no vaccine that is effective in all age groups and against all serogroups, despite extensive research efforts [2] [3]. Candidate antigens for inclusion in a vaccine must be expressed in the majority of strains, be antigenically conserved, and elicit a protective immune response. The sequencing of the genomes of a number of meningococcal strains has facilitated the identification of novel antigens by a bioinformatic approach [4]. Analysis of genes up-regulated after contact with epithelial cells [5] or endothelial cells or serum [1] using micro-arrays has identified more potential vaccine candidates. This is in addition to all the protein and carbohydrate antigens investigated prior to genome sequence data became available [3]. In previous work we reported the sequence of NhhA in *N. meningitidis* [6], which is a homologue of the adhesins Hia and Hsf of *Haemophilus influenzae* [7], [8], [9]. NhhA has also been referred to as Hsf [10] and Msf [11]. This protein prevents complement attack [12] by binding vitronectin [11], and purified NhhA stimulates a proinflammatory response [13]. NhhA is a strong vaccine candidate because of the ubiquitous presence of the *nhhA* gene in meningococcal strains, its surface exposure and the high level of sequence conservation between strains (amino acid identity 85.3%–99.8%; [6]). The majority of the sequence variation that does exist is limited to four distinct variable regions (V1–V4) located in the first 200 amino acids of the mature protein [6]. Unlike many other outer membrane proteins of *N. meningitidis*, there are no obvious sequence features such as short tandem DNA repeats in or upstream of the gene that may mediate phase variable expression of NhhA. Furthermore, this protein is recognized by antisera from patients [14] [15], implying that it is expressed and immunogenic *in vivo*. We now report further investigations of NhhA as a vaccine candidate, by characterizing the murine antibody response against a wild-type NhhA, and against truncated forms of NhhA and show that removal of the region of most variation does not prevent production of bactericidal antibodies.

## Materials and Methods

### Bacterial culture

*Escherichia coli* were cultured in LB media, and *N. meningitidis* were cultured on BHI agar overnight at 37°C. Kanamycin and ampicillin were added at 100 µg/mL. Tetracycline was added at a final concentration of 300 µg/mL and 15 µg/mL to select *E.coli* and *N. meningitidis* respectively. Strains used in this study are listed in table 1.

**Table 1:** Strains and plasmids used in this study.

Bacterial strains:	Genotype/relevant characteristic	NhhA phenotype	Reference
PMC21	*N. meningitidis*, serogroup C	Expresses wild-type NhhA_PMC21_	This study
¢3	*N.meningitidis*, derived from MC58, acapsulate (Δ*siaD::erm*), Opa - variant	Expresses wild-type levels NhhA_MC58_	[23]
2A	*N.meningitidis*, ¢3 derivative, *nhhA::kan*	NhhA expression abolished	[6]
¢3*lgtA*	¢3 derivative, *lgtA::kan*	Expresses wild-type levels of NhhA_MC58_	[18]
7G2	*N.meningitidis*, ¢3*lgtA* derivative LOS phenotype fixed L8	Expresses wild-type levels of NhhA_MC58_	This study
P6	7G2 derivative, *porA* replaced by *nhhA_PMC21_*	Over-expression of NhhA_PMC21_, wild-type levels of NhhA_MC58_	This study
P6ΔOpcA	P6 derivative. *Δopc*	Over-expression of NhhA_PMC21_, wild-type levels of NhhA_MC58_	This study
PΔ5	7G2 derivative, *porA* replaced by *nhhA_Bgl_*	Over-expression of NhhA*_Bgl_*, (Bgl II deletion) wild-type levels of NhhA_MC58_	This study
PΔ5ΔOpcA	PΔ5 derivative, *Δopc*	Over-expression of NhhA*_Bgl_*, (Bgl II deletion) wild-type levels of NhhA_MC58_	This study
PSO1	7G2 derivative, *porA* replaced by *nhhA_SO1_*	Over-expression of NhhA_SO1_, (splice-overlap deletion) wild-type levels of NhhA_MC58_	This study
PSO1.17A	PSO1 derivative, *Δopc::tet*	Over-expression of NhhA_SO1_, (splice-overlap deletion) wild-type levels of NhhA_MC58_	This study
**Plasmids**			
pC014K	*porA* gene with kanamycin resistance gene cloned downstream		[24]
pIP52(PMC21)	*nhhA_PMC21_* cloned into pC014K		This study
pIP52(PMC21Bgl)	pIP52(PMC21) derivative: *Bgl*II deletion of *nhhA_PMC21_*		This study
pIPSO1	Splice overlap deletion of *nhhA_PMC21_* cloned into pC014K		This study
pBE501	Plasmid contains *opc* gene		[16]
pIP14	*opc* deletion plasmid: *Hind*III/*Sty*I digest of pBE501		This study
pOpcTet	pBE501 derivative, *opc::tetM*		This study
pT7lgtAG7			This study
pMJ1b11	Contains *lgtABE* locus		[18]

### Over expression constructs

**Figure 1:**
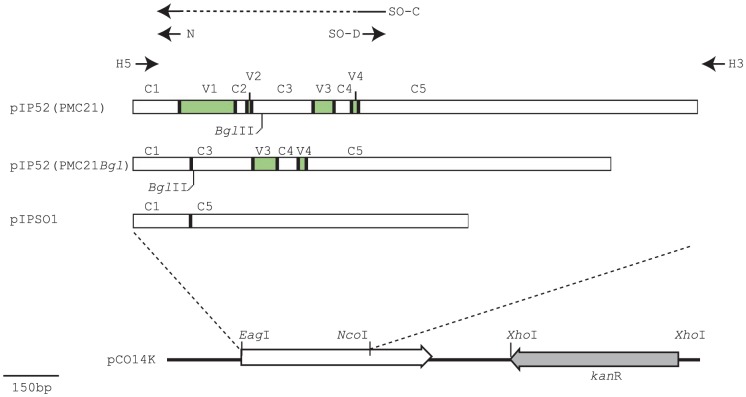
Generation of overexpression constructs. pIP52(PMC21): *nhhA*_PMC21_ was amplified using primers HOMP5' (H5 in this figure) and HOMP3'AN (H3), and cloned into the *Eag*I/*Nco*I sites of pCO14K. pIP52(PMC21Bgl): the C1 region was amplified using primers H5 and NH3'BG (N, incorporating a *Bgl*II site) and cloned into the *Eag*I/*Bgl*II sites of pIP52(PMC21). pIPSO1: the C1 region was amplified from pIP52(PMC2) using primers H5 and SO-C, and the C5 region was amplified using primers SO-D and H3. The two products were annealed, then amplified using primers H5 and H3. The resulting amplimer was cloned into the *Eag*I and *Nco*I sites of pCO14K. Conserved regions (C1–5) and variable regions (V1–V4) as defined in (19).

Plasmids used in this study are listed in table 1. To overexpress a wild-type protein, the *nhhA*_PMC21_ gene (accession number AF157611) was amplified using primers HOMP5' and HOMP3'AN (table 2). The amplimer was digested with *Eag*I and *Nco*I restriction endonucleases, and ligated into pCO14K, generating pIP52(PMC21) (Fig. 1). To overexpress a protein with most of the interstrain variable region deleted, products were amplified from chromosomal DNA or PMC21 using the primer pair HOMP5' and NH3'BG. The amplimer was digested with *Bgl*II and *Nco*I, and cloned into pIP52(PMC21) digested with *Bgl*II and *Nco*I. The resultant plasmid was named pIP52(PMC21Bgl) (Fig. 1)

**Table 2:** Oligonucleotides used for generation of variant NhhA, and lgtA amplifications.

Oligonucleotide		
HOMP5':	5′-CAA TTA ACG GCC GAA TAA AAG GAA GCC GAT **ATG AAC AAA ATA TAC CGC ATC**-3′;	This contains a *Eag*I restriction site (underlined) and the sequence encoding the first 7 (seven) amino acids of NhhA (bold type)
HOMP3'AN	5′-TGG AAT CCA TGG **AAT CGC CAC CCT TCC CTT C**-3′	This contains a *Nco*I restriction site (underlined) and the reverse complement of sequence 48–61 nucleotides past the end of the *nhhA* open reading frame of PMC21 (bold type)
NH3'BG:	5′-GGT CAG ATC TGT **TTC ATT GTT AGC ACT TGC**-3′	This contains a *Bgl*II restriction site (underlined) and the reverse complement of sequence encoding amino acids 134, (double underlined) and 49–54 of wild-type PMC21 NhhA (bold type).
SO-C	5′-GAC GAA ATC AAC GTT **CTT AGC ACT TGC CTG AAC CGT TGC**-3′.	Reverse complement of sequence encoding amino acids 237–241 at the start of the C5 region (underlined) and amino acids 45–52 at the end of the C1 region (bold type) of wild-type NhhA of strain PMC21.
SO- D:	5′-AAC GTT GAT TTC GTC CGC ACT TAC-3′	encodes amino acids 237–244 at the start of C5 (underlined indicates reverse complement of Primer SO-C)
Lic31ext	5′- CCT TTA GTC AGC GTA TTG ATT TGC G –3′	Used to mutate poly-G tract of *lgt*A and to amplify mutant *lgtA* for transformation
lgtAG3	5′-ATC GGT GCG CGC AAT ATA TTC CCC CCC GA CTT TGC CAA TTC ATC – 3′	Used to mutate poly-G tract of *lgt*A
Lic16ext	5′- CGA TGA TGC TGC GGT CTT TTT CCA T -3′	To amplify mutant *lgtA* for transformation

Splice-Overlap PCR (8,9) was used to generate pIPSO1: Oligonucleotide primers HOMP5' and SO-C were used to amplify constant region 1 (C1) and primers SO-D/HOMP3'AN to amplify constant region 5 (C5). Primers SO-C and SO-D contain complementary sequences. The two products were annealed and subsequently re-amplified using primers HOMP5' and HO3'AN. The resulting amplimer, encoding amino acids 1–52 and 337–591 of wild-type NhhA of PMC21, was digested with *Eag*I and *Nco*I, and ligated into pCO14K, generating plasmid pSO1.

### Unmarked and marked deletion of opc

The plasmid pBE501 contains the *opc* gene and flanking regions [16]. *Hind*III cuts pBE501at −41 relative to ATG start, *Sty*I cuts 570 bp downstream of that (gene is 861 bp). pBE501 was digested with *Hind*III/*Sty*I, blunted, and self ligated, deleting the majority of the gene including the start codon and part of the promoter region. The resulting plasmid was named pIP14. To create a marked deletion, pBE501 was digested with *Sty*I, deleting 327 bp, and the tetracycline resistance determinant was excised from pGEMTetA [17] and cloned into the blunted *Sty*I-digested pBE501. The resulting plasmid was named pOpcTet.

### Fixing LOS expression

In order to fix the expression of the phase variable *lgtA* gene to “off”, so that the L8 immunotype was expressed, the homopolymeric tract of the *lgtA* gene was altered so that only 7 G residues remained in the homopolymeric tract region. This results in a frame shift mutation and no expression of LgtA activity (the wild type strain, MC58, has 14 G; [18]). Using primers Lic31ext and lgtAG7 in PCR with *Neisseria meningitidis* strain MC58 chromosomal DNA as template the region encompassing the poly-G tract to be altered was amplified. The lgtAG7 primer incorporated the change in the *lgtA* sequence from 14G to 7G. The resulting amplimer was cloned into pT7Blue (Novogen), to create plasmid pT7lgtAG7. To reconstitute the complete *lgtA* gene so that the plasmid could be used to transform the new allele into *Neisseria meningitidis*, a *Bss*HII fragment from plasmid pMJ1b11 [18] was cloned into the BssHII site of pT7lgtAG7 in the correct orientation. Nucleotide sequence analysis confirmed the correct orientation of the gene and that the sequence segment was identical to the corresponding section of the wild-type *lgtA* gene apart from the alteration of the homopolymeric tract from 14 to 7 G residues. In order to transfer the *lgtAG7* mutation to the chromosome of *Neisseria meningitidis* to make a mutant strain, the plasmid pT7lgtAG7 was linearized and used to transform *Neisseria meningitidis* strain ¢3*lgtA* (containing an *lgtA::kan* mutation, [18]). Confirmation of the transfer of the *lgtAG7* allele to the chromosome in kanamycin sensitive colonies obtained from the transformation was confirmed by PCR of the relevant section of the *lgtA* gene using primers Lic31ext and Lic16ext, followed by nucleotide sequencing with the same set of primers.

### Nm transformation and screening

The plasmids were linearised by restriction digestion and used to transform *N. meningitidis* using the method described by Janik *et al*. [19]. Transformants were selected by overnight incubation at 37°C in 5% CO_2_ on solid media containing antibiotic as appropriate.

### Screening for NhhA overexpression and other protein electrophoresis

Following transformation with linearised pIP52(PMC21), pIP52(PMC21Bgl), or pIPSO1, kanamycin resistant colonies were selected, subcultured overnight and screened for over-expression of NhhA by separating total cell proteins electrophoretically on duplicate 10% acrylamide gels, followed by coomassie Blue staining or were Western blotted to nitrocellulose (BioRad) before block in skim milk/PBST followed by sequential incubation in rabbit polyclonal anti-NhhA sera [6], goat anti-rabbit alkaline-phosphatase, and detection with NBT/BCIP substrate (BioRad). The *nhhA* allele was sequenced to confirm replacement of *porA* with the PMC21 or truncated allele. For observing overexpression, sarkosyl-insoluble proteins were separated electrophoretically using Bis-Tris buffer system and 4–12% precast 8 cm gels with MOPS buffer (Invitrogen), or for western immunoblot proteins were separated using Tris-Acetate 3–8% pre-cast gels (Invitrogen).

### Screening for removal of Opc expression

Antisera containing Opc-specific polyclonal rabbit antibodies were raised by immunizing rabbits with sarkosyl-insoluble OMCs of strain P6 (Opc-expressing strain). Serum was adsorbed against strain P6ΔOpcA as previously described [20], removing most antibodies except those recognizing Opc. Following transformation with linearised pIP14 (unmarked deletion construct of *opc*), bacteria were plated at low density. After overnight incubation, colonies were transferred to nitrocellulose (BioRad) and immunoblotted using rabbit Opc-specific sera, and antibody binding was visualized using goat anti-rabbit-Alkaline phosphatase and colorimetric detection with NBT/BCIP. Colonies that had lost Opc reactivity were identified and subcultured, and analysed by immunoblot. Southern blot analysis (using DIG-labelled *Hind*III/*Sty*I fragment or *opc* gene from pBE501 as a probe) was used to confirm that the lack of expression was due to the introduced mutation and not to the inherent phase-variation of this gene, and PCR confirmed the presence of the deleted *opc* allele. Anti-Opc sera were also used to confirm loss of Opc expression following transformation with pOpcTet and selection with tetracycline.

### Protein sequencing

Sarkosyl-insoluble proteins (enriched for outer-membrane proteins) were separated by SDS-PAGE (7.5% acrylamide) and transferred to PVDF membrane. The PVDF membrane was stained with Coomassie Blue and the high molecular weight protein excised and subjected to N-terminal sequencing in a PE Biosystems 492cLC protein sequencer.

### Culture and preparation of OMVs

A vial of frozen *N. meningitidis* (recombinant or not) was thawed and streaked onto modified Frantz medium agar plate which was then incubated at 37°C for 18 h. Colonies were resuspended and added to a flask containing modified Frantz medium supplemented with the appropriate antibiotic, and incubated at 37°C for 16 h under shaking. The cells were separated from the culture broth by centrifugation at 5,000 *g* at 4°C for 15 min. OMVs were isolated using deoxycholate as described previously [21].

### Mice and immunizations

Outbred OF1 mice (Charles River, Lyon, female, 6–8 wks of age, also known as CF1) received three injections with OMVs via intramuscular route on days 0, 21 and 28. With each injection of 50 µl, 10 µg of antigen formulated onto 100 µg of Al^3+^ (aluminum hydroxide) was administered. Control mice received adjuvant only. Blood samples were collected 14 days after the third injection.

#### Ethics

Mice were provided with food and water ad libitum and were monitored for adverse events following injections. Mice were anaesthetized prior to collection of blood from carotid artery prior to cervical dislocation under continuing anaesthesia. The experiments have complied with the relevant national guidelines of Belgium and institutional policies of GlaxoSmithKline Biologicals and all protocols were approved by GSK Biologicals ethics committee.

### Antibody assays

The NhhA derived from strain H44/76 was expressed and purified from *E. coli* as C-terminally His tagged protein. ELISA plates were coated with NhhA. The assays were performed as described previously [22].

### Complement-dependent bactericidal antibody assays

Bactericidal assays were performed as described previously [10]. Briefly, bacteria from an overnight on Mueller Hinton agar plates were inoculated in tryptic soy broth with iron chelator and grown in shaking flasks for 3 h at 37°C. The culture was diluted in order to reach an OD_600 nm_ of 0.4 (bacterial suspension). The sera were heat inactivated (40 min at 56°C) and subsequently diluted in PBS-glucose. In microtiter plates, diluted test serum was mixed with baby-rabbit complement and bacterial suspension. Serial dilutions of test sera were treated similarly. Controls included bacteria plus complement, bacteria plus heat-inactivated complement, and test serum plus bacteria plus heat-inactivated complement. The microtiter plates were sealed and incubated while shaking (520 rpm) for 75 min at 37°C without CO_2_. Agar was added to each well and after an overnight incubation at 33°C in 5% CO_2_, the colonies were counted. Bactericidal titers are defined as the reciprocal of the serum dilution yielding 50% killing. Additive or synergistic effects of antibodies directed against different over-expressed minor OMPs were studied by using sera pooled from mice immunized with each vaccine preparation. For mixing experiments, equal volumes of pooled sera of each relevant treatment group were combined and subsequently tested in the bactericidal assay.

## Results

### Expression Strains

In order to remove confounding factors in analysis of immune responses to NhhA, a number of phase-variable and/or immunodominant components of the meningococcus were fixed or abrogated: the starting strain was ¢3, an acapsulate, Opa-negative derivative of serogroup B strain MC58 [23]. Phase variation of lacto-*N*-neotetraose in LOS was abolished by a making an unmarked frameshift mutation in the *lgtA* gene, resulting in a strain expressing an L8 immunotype LOS structure. Transformation with the NhhA overexpression constructs resulted in deletion of *porA*, and subsequent manipulation (as described in material and methods, and below) abolished Opc expression.

### NhhA Overexpression constructs

Our previous observations suggested that NhhA is not strongly expressed *in vitro*, so plasmids were constructed containing alleles to express NhhA at high levels when introduced into the *N. meningitidis* chromosome. As *nhhA* exhibits some sequence variation, (mostly confined to the region encoding the predicted amino-terminal 200 amino acids of the predicted mature protein), alleles were also constructed with some or all of this region deleted (See materials and methods).

The strategy of gene replacement was used: the recombinant *nhhA* allele was placed under the control of the strong *porA* promoter, using plasmid pCO14K. The plasmid pCO14K contains *porA* (promoter and coding region) with the selectable kanamycin resistance determinant downstream [24]. Alleles of *nhhA* were cloned into convenient restriction sites in pCO14K downstream of the *porA* promoter, replacing the majority of the *porA* gene (Fig. 1). Transformation of *N. meningitidis* with these plasmids results in reciprocal exchange by homologous recombination resulting in expression of the *nhhA* gene under the control of the *porA* promoter, and abrogation of *porA* expression.

For overexpression of the wild-type allele, *nhhA* was amplified from serogroup C strain PMC21 and cloned to create plasmid pIP52(PMC21). Plasmids with deletions of the most variable regions were also constructed. Plasmid pIP52(PMC21Bgl) contains a recombinant allele of *nhhA*, encoding amino acids 1–54 and 134–591 of NhhA_PMC21_, a truncation of 81 amino acids relative to wild-type. This amino acid sequence, NhhA_Bgl_, has the majority of the V1 region (most variable), all of the V2 and C2 regions, and part of the C3 region removed relative to the parental NhhA_PMC21_ protein (Fig. 1). Plasmid pIPSO1, constructed by a PCR strategy, encodes a further truncated allele, *nhhA_SO1_*, encoding amino acids 1–52 and 337–591 (*i.e.* lacking 285 amino acids relative to wild-type NhhA_PMC21_) with V1–V3 and C2–C4 deleted. (Fig. 1).

### Transformation and Over-Expression analysis

The plasmids pIP52(PMC21), pIP52(PMC21Bgl), and pSO1 were linearised and individually transformed into *N. meningitidis* strain 7G2 (acapsulate, Opa^−^ L8 immunotype fixed expression, see table 1). In each case, approximately 20–30 kanamycin resistant clones were screened to find one clone in which the double cross-over integration event had occurred to include both the kanamycin resistance determinant and the recombinant *nhhA* allele, replacing *porA* and expressing NhhA at a higher level compared with the parental strain. To confirm overexpression of NhhA and replacement of *porA* in resulting kanamycin resistant clones, outer-membrane proteins were prepared and separated electrophoretically, prior to visualization of proteins by coomassie stain.

### Deletion of opc

In order to further reduce confounding factors in our analysis of the vaccine potential of NhhA, we abolished Opc expression from our strains. Plasmids pIP14 and pOpcTet contain a deleted allele of *opc*, the latter with insertion of *tetM* resistance cassette (see methods). Opc is an outer membrane protein that is immunodominant, but is not present in all strains [25]. Plasmid pIP14 or pOpcTet was linearised and transformed into strains over-expressing NhhA variants. Colonies were screened by immunoblot using Opc-specific polyclonal rabbit sera (results not shown). Colonies that did not express Opc were analysed by immunoblot, PCR, and Southern blot to confirm that the lack of expression was due to the introduced mutation and not to the inherent phase-variation of this gene (results not shown). Thus strains used for immunogenicity studies overexpressed NhhA variants, expressed no PorA or Opc, had LOS fixed in the L8 immunotype, and these characteristics were introduced into a previously defined acapsulate, Opa-negative strain [23].

### Analysis of high molecular weight NhhA species

**Figure 2:**
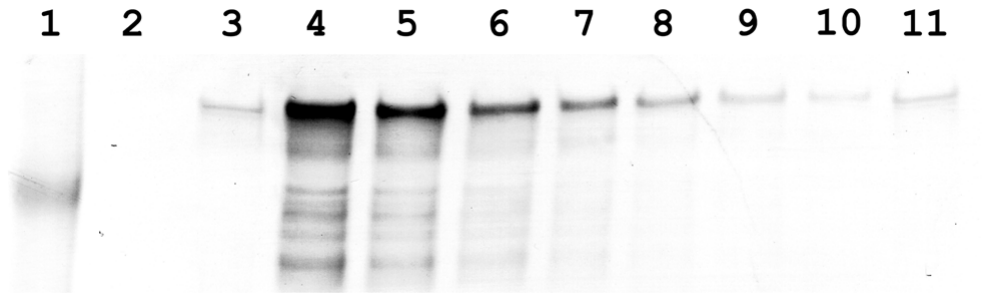
Overexpression of NhhA. Whole cell lysates were separated on 3–8% Tris-Acetate buffered PAGE, transferred to nitrocellulose, and immunoblotted with rabbit polyclonal anti-NhhA sera. Lane 1 Benchmark Prestained Marker, approximately 180 kDa marker indicated. Lane 2, 25 µg protein of strain 2A (NhhA expression abolished); Lanes 3 and 11, 25 µg protein of strain 7G2 (parental strain expressing wild type levels of NhhA), Lane 4, 25 µg protein of strain P6ΔOpcA. Lane 5, 12.5 µg protein of strain P6ΔOpcA; Lane 6, 6.25 µg protein of strain P6ΔOpcA; Lane 7 3.125 µg protein of strain P6ΔOpcA; Lane 8, 1.56 µg protein of strain P6ΔOpcA; Lane 9, 0.78 µg protein of strain P6ΔOpcA; Lane 10, 0.39 µg protein of strain P6ΔOpcA.

As NhhA is a surface expressed protein [6], each of the overexpressed NhhA proteins were engineered to include the signal peptide (amino acids 1–51) predicted by SIGNALP (not shown). In each of the resulting strains, NhhA expression upregulated relative to the non-transformed parental strain, and PorA expression was abolished. In order to determine the level of recombinant NhhA overexpression relative to the endogenous NhhA, serial two-fold dilutions and Western immunoblot of sarkosyl insoluble proteins was conducted with detection by rabbit polyclonal anti-NhhA sera [6]. Expression of NhhA is increased at least 25-fold (Fig. 2) for the full-length NhhA-overexpressing strain P6ΔOpcA. Reactivity with NhhA-specific sera is equivalent between 25 µg sarkosyl-insoluble protein of strain 7G2, and <1 µg protein of strain P6ΔOpcA.

**Figure 3:**
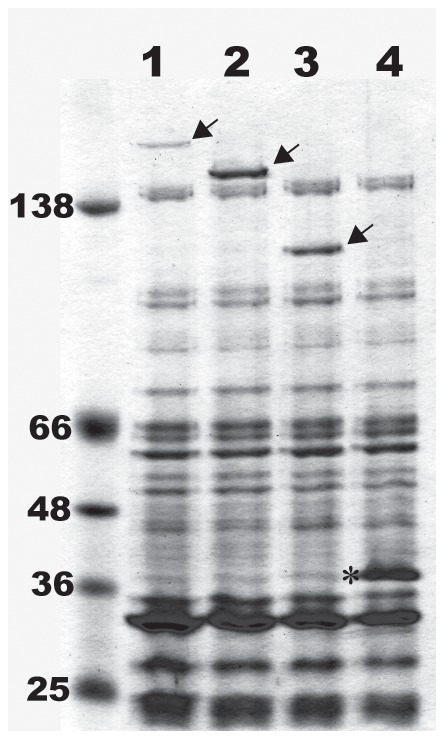
Overexpression of NhhA variants. Sarkosyl-insoluble fractions were separated electrophoretically on 4–12% gradient Bis-Tris acrylamide gels and Coomassie stained. Lane 1: Strain P6, Lane 2: Strain PΔ5, Lane 3: Strain PSO1, Lane 4: Strain ¢3. Arrowheads indicate NhhA_PMC21_ (lane1), NhhA_Bgl,_(lane 2), and NhhA_SO1_ (lane 3). Asterisk indicates position of PorA. Apparent MW of ColorPlus markers indicated. See Table 1 for strain and protein descriptions.

It is possible that the high molecular weight species expressed by NhhA overexpressing strains represent either stable multimers of NhhA (Fig. 3), or complexes of NhhA with other protein(s), or other proteins alone. To confirm the identity of the high molecular weight protein, and to confirm the cleavage of the predicted signal peptide, the overexpressed proteins were N-terminally sequenced. Amino acid sequence for the two truncated NhhA proteins indicated in each case that the high molecular weight complex is comprised essentially of NhhA. The N-terminal sequence XXETDLTSVGT was obtained for protein isolated from PΔ5ΔOpcA, which corresponds to predicted amino acids 52 to 62 of the truncated non-mature allele. The N-terminal sequence XNVXFVRTY was obtained for the PSO1.17A-derived protein, corresponding to amino acids 52–60. These data confirm the presence and cleavage site for the long signal peptide (aa 1–51), and that it is cleaved when expressed in *N. meningitidis*. The predicted molecular weight of the mature forms (*i.e.* after cleavage of the predicted signal peptide) of NhhA_PMC21_, NhhA_Bgl_, and NhhA_SO1_, is 56.6, 47.6, and 36.7 kDa respectively. We had previously noted that NhhA migrates on SDS-PAGE at a size equivalent to >250 kDa [6] and NhhA has recently been described as a trimer when expressed in *E. coli* [26]. Overexpression in *N. meningitidis* of NhhA_PMC21_, and of the truncated proteins NhhA_Bgl_, and NhhA_SO1_, results in migration at a size implying that NhhA exists as a stable mulitimeric form (Fig. 2) and that the truncations did not remove any structural features necessary for oligomerisation or stability.

### Immunogenicity

**Figure 4:**
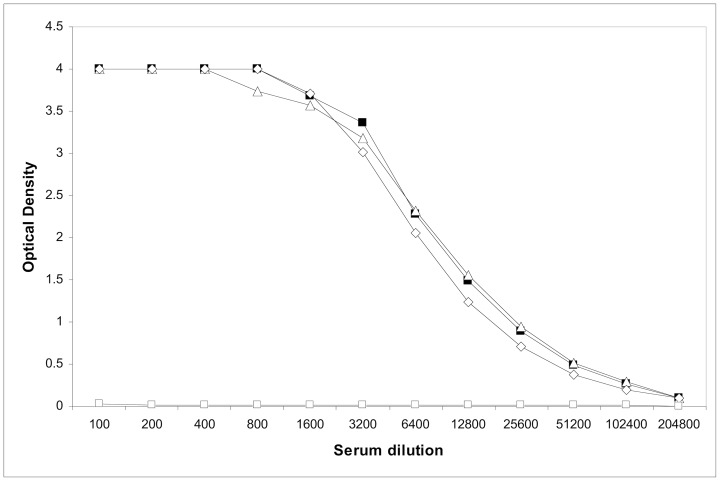
ELISA response in immunized mice. Pooled sera from mice immunized with OMVs purified from strains over-producing either the wild type NhhA_PMC21l_ (black square), the NhhA lacking variable loops 1&2 (NhhA_Blg_, open triangle), or the NhhA without the four variable regions (NhhA_SO1_, open diamond), and from control mice (open square) immunized with adjuvant alone. Target antigen was recombinant NhhA purified from expression in *E. coli*.

In order to assess the antibody response to the NhhA variants, outer membrane vesicles were prepared. The OMV preparations were adsorbed onto Al(OH)_3_ and injected into mice on days 0, 21 and 28. On day 42, the mice were bled and sera pooled from animals in each group. Each vaccine preparation elicited a similar antibody response, as assessed by ELISA using purified recombinant NhhA_H44/76_ (Fig. 4). Mice immunized with OMV produced from a NhhA null strain did not elicit anti-NhhA antibodies (data not shown).

As it has previously been observed that over-expression of more than one minor protein is required for high-titer bactericidal activity [10], sera from mice inoculated with OMVs obtained from strains over-expressing the different NhhA variants (NhhA-OMVs) were mixed with sera from mice immunized with OMVs produced from a TbpA over-expressing strain (TbpA-OMVs) [10] and serum bactericidal assays were performed against H44/76 and CU385 (Table 3).

**Table 3:** Impact of deletion of variable regions of Nhha on the induction of complement-mediated killing by bactericidal antibodies in mice in synergy with anti-TbpA OMVs sera using pooled sera.

Mix of sera^b^	H44/76 SBA^a^	CU385 SBA^a^
a-TbpA OMVs+control negative pooled sera	778	532
a-TbpA OMVs+a- NhhA_PMC21_ OMVs	2595^a^	1438
a-TbpA OMVs+a- NhhA_Bgl_ OMVs	4383	2891
a-TbpA OMVs+a- NhhA_SO1_ OMVs	1568	742

a SBA = Serum Bactericidal Assay, Geometric mean titers for 50% killing.

b NhhA_PMC21_, wild type NhhA; NhhA_Bgl_, NhhA without variable regions 1&2; NhhA_SO1_, NhhA without variable regions 1 to 4.

The mix of pooled sera from mice immunized with TbpA-OMVs and NhhA_PMC21_-OMVs had higher bactericidal titers than mixed sera from control mice (immunized with adjuvant alone) and from mice immunized with TbpA-OMVs. These results are in line with results obtained previously showing additive or synergistic impact of antibodies against more than one minor component for bactericidal activity [10]. The highest bactericidal titers were measured on the mix of anti-TbpA-OMV sera and NhhA_Bgl_-OMVs (NhhA_Bgl_ lacking the variable regions V1 and V2). The enhanced bactericidal activity of NhhA_Bgl_ was seen against both the strains used in SBA. By comparison, the deletion of the four variable regions of NhhA (NhhA_SO1_) clearly had a negative impact on the induction of bactericidal antibodies as the bactericidal titers measured on the mixed anti-TbpA sera were lower than those obtained either with the wild type NhhA_PMC21_ or with NhhA_Bgl_.

## Discussion

Many outer membrane proteins of *N. meningitidis* exhibit strain to strain sequence variation, presumably as a result of selection by the host immune system. This is one of the reasons no effective cross-protective vaccine is available. The most variable region of PorA, for example, are in the longest, surface exposed loops which are highly immunogenic, and in part responsible for the strain specificity of previous vaccine formulations. NhhA has a number of variable regions, but the longest and most variable region (V1) is confined to the amino-terminus of the mature protein ([6], Fig. 1). It has been previously noted that sera from meningococcal disease patients recognize NhhA. Van Ulsen *et al.* [14] suggest the reactivity of patient sera implies that cross-reactive antibodies are elicited *in vivo* despite the infecting strain being different in each case [14]. Another study indicated that convalescent sera of meningococcal disease patients (age 0.2–4 yrs) also recognises NhhA (referred to as NMB0992, [15]). However, whether the antibodies were bactericidal, and the allele of *nhhA* in the infecting strain was not investigated. Healthy carriers also had antibodies that recognized NhhA [14], which may be the result of asymptomatic colonisation by *N. meningitidis*. NhhA expression levels are variable between strains [6], [27], but the mechanisms for this variable expression are unclear.

We over-expressed the wild-type protein to assess immunogenicity and protective potential against the autologous strain. We also overexpressed two truncated alleles to assess whether the conserved regions could elicit a protective immune response. As a consequence of the method of over-expression, these strains were deficient for PorA production. In addition, we deleted the strongly immunogenic protein Opc, in a strain background that was acapsulate, Opa^−^, and phase variation of terminal LOS was abolished. The resulting phenotype, lacking several immundominant OMPs enabled assess the potential of NhhA for inclusion in a vaccine.

As NhhA is expressed at low levels *in vitro* in many strains, we placed NhhA under the control of the *porA* promoter resulting in strong expression of NhhA, as previously reported [10]. NhhA shares sequence similarity with other autotransporter proteins [6], particularly the adhesins Hia and Hsf of *H. influenzae*. One of the features of autotransporters is a long signal peptide: we confirmed that the mature protein has a signal peptide that is cleaved after amino acid 51. We also observed that for each of the overexpressed NhhA proteins and truncations, NhhA migrated on SDS-PAGE at much greater than the predicted molecular weight. This demonstrated that removal of N-terminal domains did not affect trimerisation and stability. The stability and correct trimerisation of the recombinant NhhA described in this study is important, as expression of monomeric NhhA (due to mutation of the translocator domain) results in reduced susceptibility to NhhA-specific bactericidal killing [27].

The autotransporters YadA (*Yersinia* sp.) and UspA1 (*Moraxella catarrhalis*) form a membrane anchored oligomeric “lollipop” [28] and the C-terminal 76 amino acids of Hia form a trimer in the outer-membrane and are necessary and sufficient for export of the passenger domain [29]. Surana *et al.* also showed that the C-terminal 119 amino acids of NhhA could act to surface localize the Hap passenger domain [29]. More recently, Scarselli *et al.*, demonstrated that the transporter domain of NhhA could be further localized to the C-terminal 72 amino acids [26].

By analogy with the structure of related autotransporters such as YadA [30], the N-terminal domain of the mature NhhA protein is predicted to form the tip region of a filamentous structure, and the most variable region of *nhhA* encodes this N-terminal domain. The deletion of variable regions (V1 to V2 or V1 to V4) did not reduce the immunogenicity of the truncated variants when presented in OMVs obtained from NhhA over-expressing strains, as assessed by ELISA to wild-type recombinant NhhA. This indicates that proteins of the OMVs are processed in such a way to elicit antibodies to epitopes other than the variable regions, and that these epitopes are accessible on recombinant *E. coli*-derived NhhA. Previous studies using another vaccine candidate, FrpB, also demonstrated that removal of immunogenic domains does not reduce production of antibodies recognizing wild-type FrpB when assessed by ELISA [31]. Crucially, in the FrpB study, the conserved cryptic epitopes were not accessible when the wild-type protein is expressed.

To assess whether antibodies raised against truncated NhhA could access the same epitope on wild-type proteins, bactericidal assays were performed. This was done by mixing anti-NhhA-OMV sera with anti-TbpA-OMV sera because it was previously observed that NhhA-OMVs alone elicited reduced or undetectable serum bactericidal antibody titers in mice [10]. When high levels of NhhA are expressed in a target strain, however, polyclonal NhhA-specific sera are bactericidal [27]. The results presented here confirmed the previous observation that antibodies directed against different minor OMPs work either in addition or in synergy to mediate the complement killing of bacteria [10], as the bactericidal titers measured on the pooled sera from mice immunized with TbpA-OMVs were systematically lower than the titers observed for the different NhhA-TbpA mixed sera.

In contrast to results obtained for FrpB truncations, removal of variable regions 1 and 2 of NhhA did not reduce bactericidal activity induced by OMVs containing the truncated NhhA_Bgl_. Indeed, the bactericidal titer induced by NhhA_Bgl_-OMVs against both tested strains was higher than that elicited by NhhA_PMC21_-OMVs expressing wild-type, full length NhhA. NhhA_H44/76_ and NhhA_CU385_, despite being geographically distinct (from epidemics in Norway and Cuba respectively) contain NhhA that differed by only one amino acid located in the signal peptide. The differences in bactericidal titers against these two strains may be explained by differences in TbpA (amino acid sequence is 92% identical), in differences in other minor antigens within the OMVs, or in levels of capsular polysaccharide produced. Nevertheless, in both cases the highest titers were with the NhhA_Bgl_-OMVs, confirming that antibodies against this truncated protein can act synergistically with anti-TbpA to mediate complement activation and killing. As removal of the variable regions did not reduce functional antibody responses, it is tempting to speculate that this truncated variant would elicit a response against strains expressing NhhA whose sequence differs within the variable domain but this remains to be formally examined.

Further truncation of NhhA (NhhA_SO1_) resulted in a reduced bactericidal response against wild-type target strains, but not a reduction in immunogenicity assessed by ELISA. A similar effect has been reported when variable loops were selectively deleted from the Envelope protein of HIV: removal of variable loops resulted in an immune response capable of neutralizing strains containing variant loops whereas further truncations abolished neutralizing activity [32]. The fact the deletion of V1 to V2 or V1 to V4 regions of NhhA in our study does not impact on the immunogenicity of the OMV vaccine (as observed in ELISA) but does affect the induction of bactericidal antibodies suggests either that some protective epitopes are localized in the C3 domains or that the larger truncation of the N terminal part of the protein impact on the conformation of the NhhA passenger domain. Another possibility is that removal of these variable domains of NhhA reveals another protein that is responsible for the synergistic protection, although this is considered less likely. It is also notable that the larger truncation of NhhA involves the first of two consensus sequences between N-CAMs and NhhA, domains which may be involved in the potential adhesin function of NhhA [33]. NhhA also inhibits complement–mediated killing via vitronectin binding [11], but which domains of the protein mediate this is currently not elucidated.

Taken together, our studies suggest that the strategy of removing the most variable region of this protein still elicits an effective antibody response. In this study, we demonstrated that an OMV vaccine incorporating NhhA with the most variable region deleted elicits an higher bactericidal antibody titer than one containing the the wild-type protein. Removing this region results in an antigen with potential to elicit responses effective against strains with variant NhhA sequence, thus truncated NhhA represents a promising candidate for inclusion in an OMV based vaccine formulation for prevention of meningococcal disease.
